# Alternative (backdoor) androgen production and masculinization in the human fetus

**DOI:** 10.1371/journal.pbio.3000002

**Published:** 2019-02-14

**Authors:** Peter J. O’Shaughnessy, Jean Philippe Antignac, Bruno Le Bizec, Marie-Line Morvan, Konstantin Svechnikov, Olle Söder, Iuliia Savchuk, Ana Monteiro, Ugo Soffientini, Zoe C. Johnston, Michelle Bellingham, Denise Hough, Natasha Walker, Panagiotis Filis, Paul A. Fowler

**Affiliations:** 1 Institute of Biodiversity, Animal Health and Comparative Medicine, College of Medical, Veterinary and Life Sciences, University of Glasgow, Glasgow, United Kingdom; 2 Laboratoire d'Etude des Résidus et Contaminants dans les Aliments (LABERCA), UMR Oniris-INRA 1329, Université Bretagne Loire, Nantes, France; 3 Department of Women's and Children's Health, Pediatric Endocrinology Unit, Karolinska Institute and University Hospital, Stockholm, Sweden; 4 Institute of Medical Sciences, School of Medicine, Medical Sciences and Nutrition, University of Aberdeen, Aberdeen, United Kingdom; University of Cambridge, UNITED KINGDOM

## Abstract

Masculinization of the external genitalia in humans is dependent on formation of 5α-dihydrotestosterone (DHT) through both the canonical androgenic pathway and an alternative (backdoor) pathway. The fetal testes are essential for canonical androgen production, but little is known about the synthesis of backdoor androgens, despite their known critical role in masculinization. In this study, we have measured plasma and tissue levels of endogenous steroids in second trimester human fetuses using multidimensional and high-resolution mass spectrometry. Results show that androsterone is the principal backdoor androgen in the male fetal circulation and that DHT is undetectable (<1 ng/mL), while in female fetuses, there are significantly lower levels of androsterone and testosterone. In the male, intermediates in the backdoor pathway are found primarily in the placenta and fetal liver, with significant androsterone levels also in the fetal adrenal. Backdoor intermediates, including androsterone, are only present at very low levels in the fetal testes. This is consistent with transcript levels of enzymes involved in the alternate pathway (steroid 5α-reductase type 1 [SRD5A1], aldo-keto reductase type 1C2 [AKR1C2], aldo-keto reductase type 1C4 [AKR1C4], cytochrome P450 17A1 [CYP17A1]), as measured by quantitative PCR (qPCR). These data identify androsterone as the predominant backdoor androgen in the human fetus and show that circulating levels are sex dependent, but also that there is little de novo synthesis in the testis. Instead, the data indicate that placental progesterone acts as substrate for synthesis of backdoor androgens, which occurs across several tissues. Masculinization of the human fetus depends, therefore, on testosterone and androsterone synthesis by both the fetal testes and nongonadal tissues, leading to DHT formation at the genital tubercle. Our findings also provide a solid basis to explain why placental insufficiency is associated with disorders of sex development in humans.

## Abstract 2

Fetal human masculinisation depends on testosterone production by the testes and an alternative “backdoor” androgen. This study shows that this androgen is likely to be androsterone, which is sexually dimorphic in the fetus but does not come from the testes; instead, synthesis probably depends on placental substrates.

## Author summary

The human penis starts to develop before birth from a structure called the genital tubercle. This process is dependent on the secretion of testosterone from the fetal testes and subsequent conversion of testosterone into dihydrotestosterone (DHT) by enzymes in the genital tubercle. Recently, an alternative "backdoor" route to the formation of DHT, which does not require testosterone, has also been shown to be essential for normal development of the human penis. In this study we provide evidence indicating that androsterone is the major backdoor androgen involved in human masculinization and that it is produced in nongonadal tissues. Steroid hormone levels were measured in the plasma of second trimester human fetuses, and testosterone and androsterone were the only androgens with higher levels in males than in females. Analysis of tissue steroid levels showed that plasma androsterone did not primarily originate from the testes but, instead, was probably formed in other tissues via metabolism of placental progesterone. These data indicate, therefore, that masculinization of the human fetus depends on steroid hormone secretion from both the testes and the placenta, and would explain why placental dysfunction is associated with disorders of sex development.

## Introduction

The male external genitalia are the most common site of congenital abnormalities in the human, with up to 0.8% of male births affected [1,2]. The most frequent of these abnormalities is hypospadias, which is characterized by abnormal opening of the urethra on the ventral side of the penis. Normal masculinization of the fetus is dependent upon androgen secretion by the testis, and androgens act initially during a critical masculinization programming window to ensure normal male reproductive development [3–5]. In humans, male-specific morphological differentiation of the genital tubercle/penis begins around 10 weeks of gestation (i.e., 8 weeks postconception), with closure of the urethral groove [6,7]. The process is complete by about gestation weeks 15–16.5 [6,7], although sexually dimorphic growth of the penis continues through the second and third trimesters [8]. The etiology of hypospadias is probably multifactorial, but it is likely that altered androgen exposure during the second trimester is a significant factor [9].

**Fig 1:**
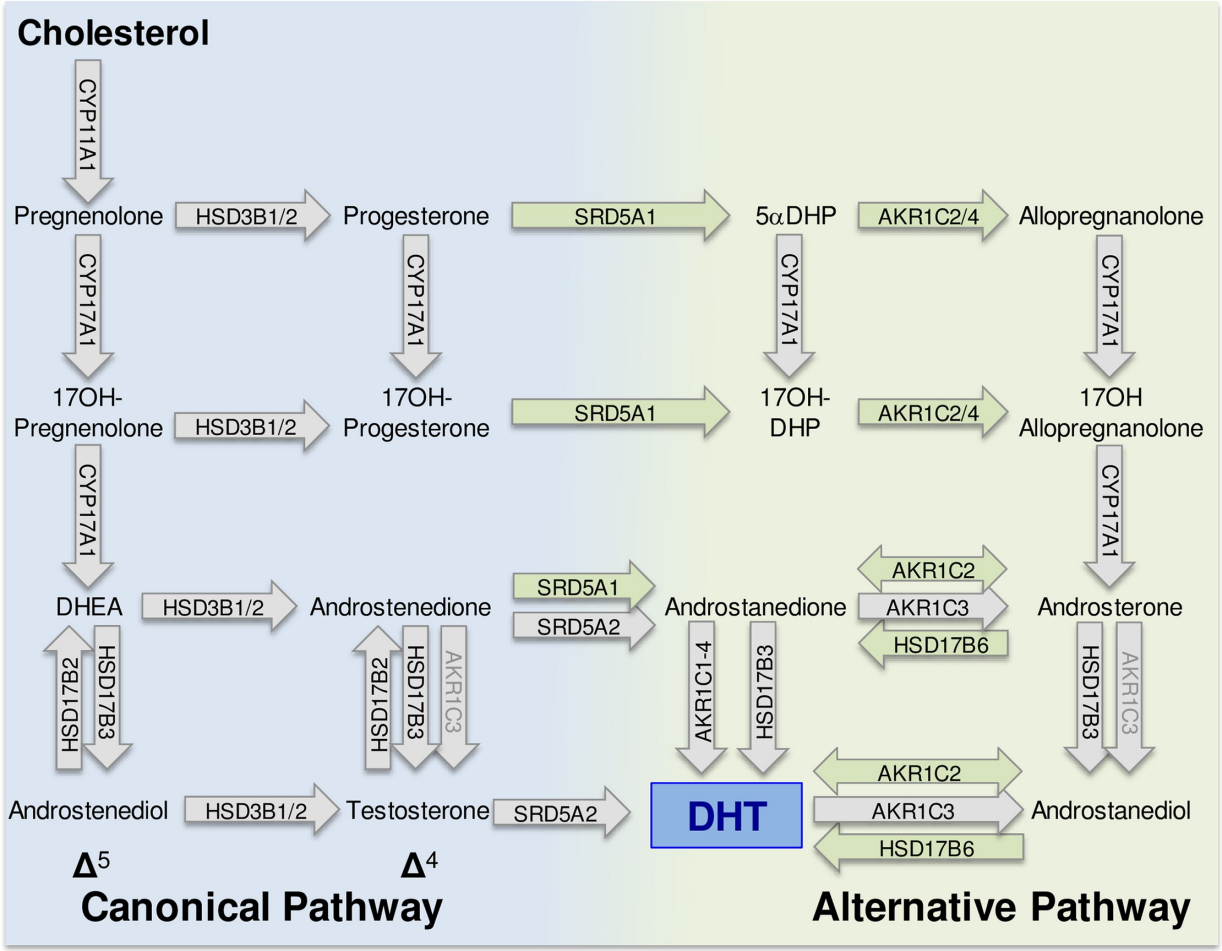
Canonical and alternative (backdoor) pathways of DHT synthesis. The canonical pathway has potential Δ^4^ and Δ^5^ subpathways. The enzymes that catalyze each step are indicated within the arrows. Enzymes written in black on a gray arrow are essential components of the canonical pathway, and some appear in both canonical and backdoor pathways (e.g., CYP17A1). Enzymes written in black on a green background are specific to the backdoor pathway. Enzymes in gray text will carry out the described conversion, but they may not be the principal enzyme involved. Other enzymes, not shown, may also be involved in components of the backdoor pathway [17]. Human CYP17A1 can convert progesterone to 17α-hydroxyprogesterone, but 17–20 lyase activity is very low with 17α-hydroxyprogesterone as substrate, and significant androstenedione is not produced by the Δ^4^ pathway in humans [18]. Similarly, 17OHDHP is a poor substrate for 17–20 lyase activity [19]. androstanediol, 5α-androstan-3α, 17β-diol; androstanedione, 5α-androstane-3,17-dione; androstenediol, androst-5-ene-3β,17β-diol; DHEA, dehydroepiandrosterone; DHT, dihydrotestosterone; 5αDHP, 5α-dihydroprogesterone; 17OHDHP, 17α-hydroxydihydroprogesterone.

During masculinization, testosterone acts directly to stabilize the mesonephric (Wolffian) ducts and to induce testis descent. However, it is conversion of testosterone to the more potent 5α-dihydrotestosterone (DHT) at the target organ that leads to masculinization of the external genitalia [10]. In humans, testosterone is synthesized in the testicular Leydig cells through the canonical Δ^5^ pathway shown in Fig 1 [11,12]. More recently, however, it has been reported that an alternative pathway to DHT formation exists that does not require testosterone as an intermediate. This alternative, “backdoor” pathway (Fig 1) was first described in the testes of pouch young marsupials [13], and a similar pathway has since been reported in the prepubertal mouse testis [14] and the fetal human testis [15]. The importance of the backdoor pathway to normal human development was initially unclear, but studies by Flück and colleagues [15] have shown that disordered sex development (DSD) will arise if the pathway is disrupted. In one individual with mutations in aldo-keto reductase type 1C2 (AKR1C2) and in another family with an added mutation in aldo-keto reductase type 1C4 (AKR1C4), there was failure of normal masculinization. Importantly, the consequences of these mutations in the backdoor pathway are similar to those seen in individuals with mutations in the canonical pathway [16]. These data demonstrate that both the canonical and backdoor pathways are essential for normal fetal masculinization.

Currently, the accepted model for masculinization is that circulating DHT, formed via the backdoor pathway in the fetal testis [15,16], is important for virilization alongside circulating testosterone. At present, however, our understanding of the regulation of human fetal masculinization is seriously hindered because we do not know which steroids are present in the male fetal circulation or fetal tissues, what the concentrations of these steroids are, whether there are sex differences, and which tissues are involved in steroid metabolism. This means that the circulating levels of DHT and potential substrates for DHT synthesis at the target organ, from either the canonical or backdoor pathways, remain unknown in the human fetus.

In this study, we have measured (i) concurrent levels of fetal plasma and tissue steroids by hyphenated mass spectrometric tools, (ii) transcript levels of critical enzymes in the backdoor pathway in human fetal tissues, and (iii) canonical and backdoor androgen synthesis by the human fetal testis in vitro. Our results show that high levels of intermediates in the backdoor pathway are present in the human fetal circulation, that androsterone is the major circulating backdoor androgen, and that female fetuses have lower levels of circulating androsterone (and testosterone). The results also show, however, that the fetal testis contains only very low levels of backdoor androgens and DHT and that androsterone is likely to be formed in nongonadal tissues, probably through metabolism of placental progesterone and adrenal dehydroepiandrosterone (DHEA).

## Results

### Significant levels of intermediates in the backdoor pathway are present in male fetal plasma, but DHT is undetectable

**Fig 2:**
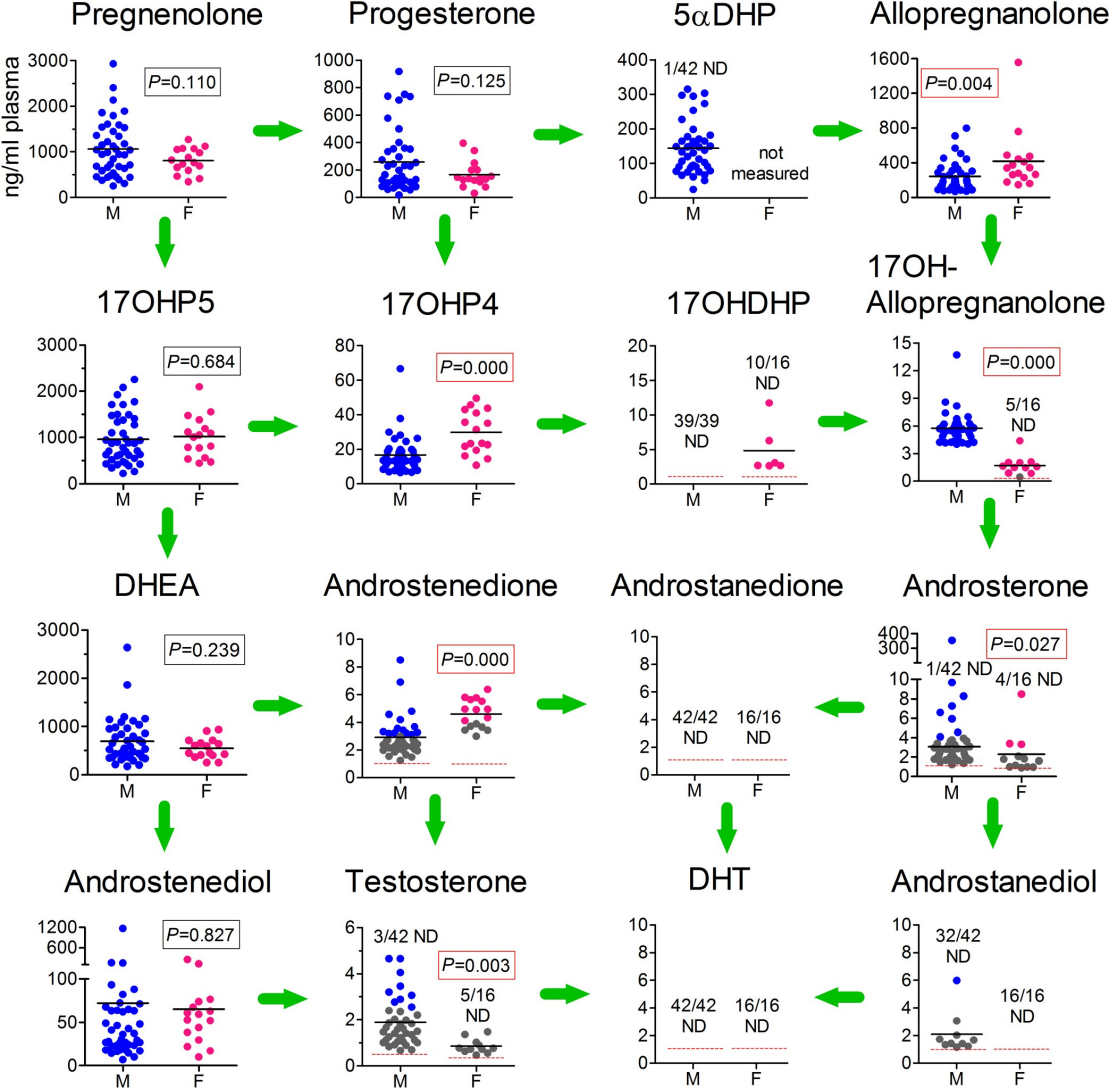
Concentrations of both canonical and backdoor steroids in the plasma of human male and female fetuses during the second trimester. Steroid levels from 39–42 individual male fetuses and 16 female fetuses (measured by GC-MS/MS) are shown in each graph, with the mean level indicated with a black line. The number of samples that were ND for each steroid are shown and, where appropriate, the LOD is shown as a red dotted line. Data shown in gray were above the LOD but below the formal LOQ, which means that the quantified data shown for these samples are less reliable than for data shown in blue or pink, which were above the LOQ. The mean for male androsterone does not include the outlier. Differences between male and female steroid levels were measured by *t* test (using the Cohen correction when appropriate), and the significance of the difference is shown on each graph. The significance value shown for androsterone was determined without inclusion of the male outlier; if the outlier is included, *P* = 0.0001 after log transformation of the data. The plasma used in these studies was from fetuses aged between 12 and 19 weeks. Steroid abbreviations used are the same as those in Fig 1. Raw data are shown in S1 Data (Sheet 1). F, female; GC-MS/MS, gas chromatography–tandem mass spectrometry; LOD, limit of detection; LOQ, limit of quantification; M, male; ND, nondetectable.

Overall levels of steroids involved in the synthesis of DHT in male fetal plasma (obtained from cardiac puncture ex vivo) are shown in Fig 2. As expected, in males all steroids in the canonical Δ^4^ and Δ^5^ pathways were present in the fetal circulation. Noticeably, the data also show that intermediates in the backdoor pathway were present at significant levels, with the backdoor pathway apparently going from progesterone through 5α-dihydroprogesterone (5αDHP), allopregnanolone, 17α-hydroxyallopregnanolone, to androsterone (Fig 2). Circulating DHT, however, was not detectable in any of the 42 male fetuses (<1 ng/mL). The Δ^5^ steroids pregnenolone, 17α-hydroxypregnenolone, and DHEA were present at the highest levels in the fetal male circulation, and these steroids probably come from the fetal adrenal gland [20,21]. Levels of progesterone were also high and were likely to be derived principally from the placenta [22] (and see below). The key initial intermediates in the backdoor pathway, 5αDHP and allopregnanolone, were present in the fetal circulation at similar concentrations to progesterone (means: progesterone, 258 ng/mL; 5αDHP, 135 ng/mL; allopregnanolone, 243 ng/mL). The principal Δ^4^ and backdoor androgens detectable in most samples were androstenedione, testosterone, and androsterone, all potential substrates for DHT synthesis. Most forms of 5α-androstanediol were undetectable in fetal plasma (including 5α-androstane-3β,17β-diol), although 5α-androstan-3α,17β-diol (labeled androstanediol in Figs 1 and 2), which is a potential substrate for DHT synthesis, was detectable in 10/42 samples (Fig 2). Etiocholanolone, a metabolite of androstenedione, was also present in most samples (S1 Fig). Levels of most steroids did not change over the course of the second trimester, with the exception of testosterone, which declined significantly (*P* < 0.048), and androstenediol, which increased (*P* < 0.004) over the same period (S2 Fig). Maternal smoking had no significant effect on fetal plasma steroid levels. A full list of steroids measured by gas chromatography–tandem mass spectrometry (GC-MS/MS) in human male fetal plasma is shown in S1 Table.

### Levels of backdoor androgens and testosterone are lower in human female fetal plasma

To place male fetal plasma steroid levels into context, the circulating steroid levels in 16 second trimester female fetuses (age matched with the 42 male fetuses) were also measured (Fig 2). Levels of Δ^5^ steroids (pregnenolone, 17α-hydroxypregnenolone, DHEA, and androstenediol) did not differ between sexes. Progesterone levels were also similar, but the Δ^4^ steroids 17α-hydroxyprogesterone and androstenedione were significantly higher in females, while testosterone was significantly lower (although there was some overlap between sexes). In the backdoor pathway, plasma allopregnanolone levels were higher in female fetuses, but 17α-hydroxyallopregnanolone and androsterone levels were significantly lower (Fig 2). Plasma levels of 5α-androstan-3α,17β-diol (androstanediol) and DHT were undetectable in all female fetuses (Fig 2), while levels of the metabolite etiocholanolone were significantly lower than in males (S1 Fig). There were no significant age-specific changes in any of the female plasma steroids measured over the course of the second trimester, although DHEA and androstenedione showed an interaction between age and maternal smoking (S3 Fig). Levels of progesterone in female fetal plasma were significantly increased by maternal smoking (S3 Fig). A full list of steroids measured by GC-MS/MS in human female fetal plasma is shown in S2 Table.

### Backdoor steroids are present primarily in nongonadal tissues in the male fetus

**Fig 3:**
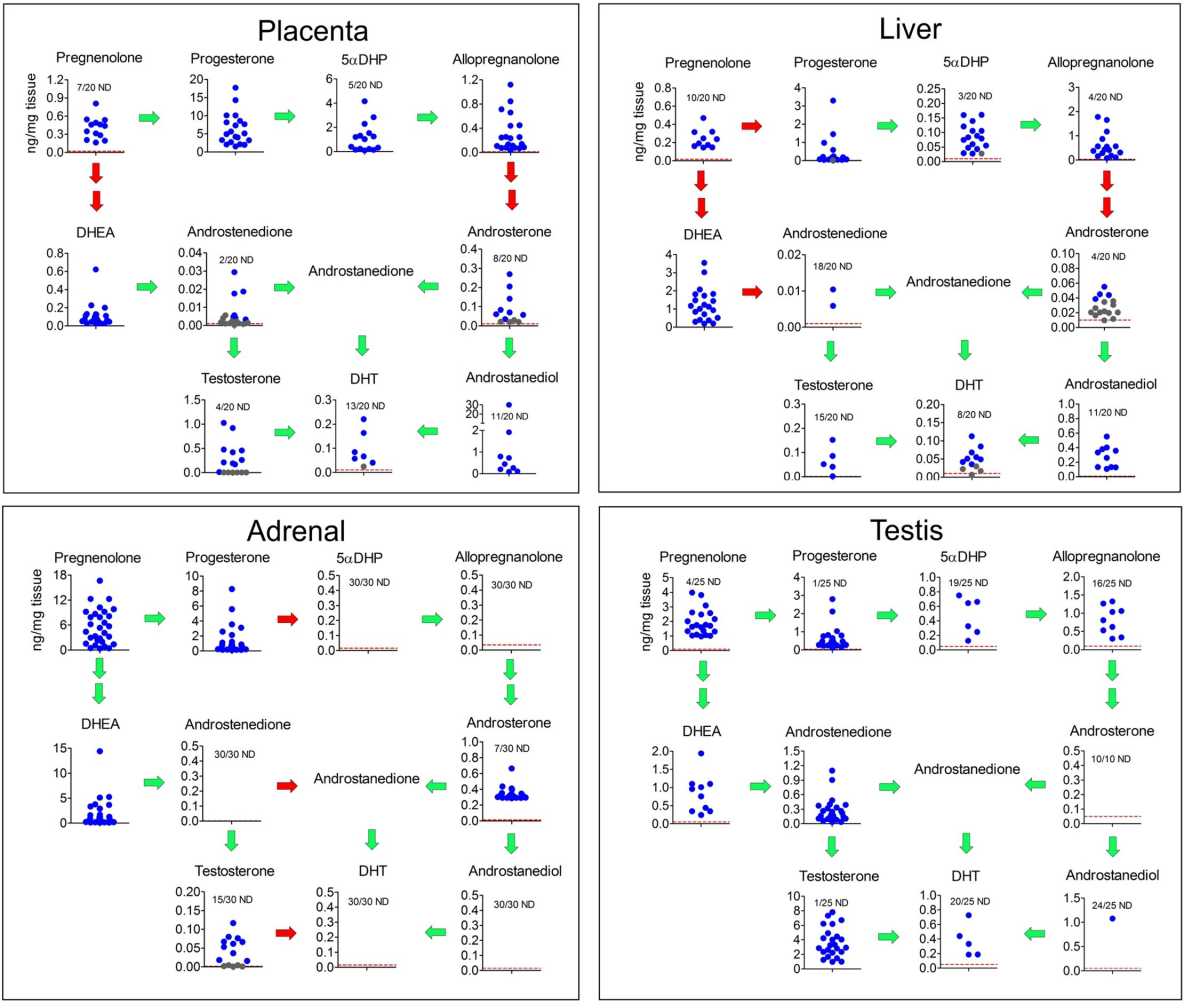
Levels of steroid intermediates involved in the canonical and backdoor synthesis of DHT in male fetal tissues. Tissue levels of steroids (measured by LC-HRMS) from the placenta and fetal liver (*n* = 20; placentas and fetal livers were from the same pregnancies), fetal adrenal (*n* = 30), and fetal testis (*n* = 10 [for DHEA and androsterone] or 25 [for all other steroids]) are shown as individual points in each graph and arranged in the pathways shown in Fig 1. Levels of 17α-hydroxylated intermediates were not measured in this part of the study. The number of samples that were ND for each steroid are shown and, where appropriate, the LOD is shown as a red dotted line. Data shown in gray were above the LOD but below the formal LOQ, which means that the quantified data shown for these samples are less reliable. The LOD for each sample (in ng/mg tissue) depended on the mass of tissue extracted, and the lines drawn are based on the average mass of each tissue used. Green arrows indicate that the relevant enzymes are detectable (as mRNA transcripts) in that tissue, while red arrows indicate that the presumed enzyme is not detectable (based on data in Fig 4). Raw data are shown in S1 Data (Sheet 2). androstanediol, 5α-androstan-3α, 17β-diol; androstanedione, 5α-androstane-3,17-dione; androstenediol, androst-5-ene-3β,17β-diol; DHEA, dehydroepiandrosterone; DHT, dihydrotestosterone; LC-HRMS, liquid chromatography–high-resolution mass spectrometry; LOD, limit of detection; LOQ, limit of quantification; ND, nondetectable; 5αDHP, 5α-dihydroprogesterone; 17OHDHP, 17α-hydroxydihydroprogesterone.

Levels of major Δ^4^, Δ^5^, and backdoor androgens in placenta, fetal liver, fetal adrenal, and fetal testis, as measured by liquid chromatography–high-resolution mass spectrometry (LC-HRMS), are shown in Fig 3. Note that 17α-hydroxylated intermediates were not measured in this study, while matrix effects meant that the 5α-reduced androgen, androstanedione, was not detectable. The placenta contained high levels of progesterone, with lower amounts of 5αDHP and allopregnanolone. The backdoor androgens, androsterone, and androstanediol were detectable in about half the placentas, while the Δ^4^ steroids androstenedione and testosterone were detectable in most placentas. DHT was also detectable in about half the placental samples (Fig 3). The major steroids detectable in the fetal liver were progesterone, allopregnanolone, and DHEA. Low levels of androsterone and DHT were also detectable in most fetal livers, while androstanediol was detectable in about half the samples (Fig 3). The fetal adrenals contained high levels of pregnenolone, progesterone, and DHEA, and androsterone was present in most adrenals. The androsterone and DHEA in the adrenals were both sulfated, which is likely to be a reflection of the high levels of sulfotransferase type 2A1 (SULT2A1) in this tissue [21]. Testosterone was present in about half the adrenals but other steroids were not detectable (Fig 3). The fetal testes contained high levels of pregnenolone and testosterone, with lower levels of progesterone and androstenedione. The backdoor intermediates 5αDHP and allopregnanolone were detectable in 6 and 9 testes, respectively (out of 25), but androsterone was not detectable, and androstanediol was only detectable in one testis. Low levels of DHT were detectable in 5 testes.

**Fig 4:**
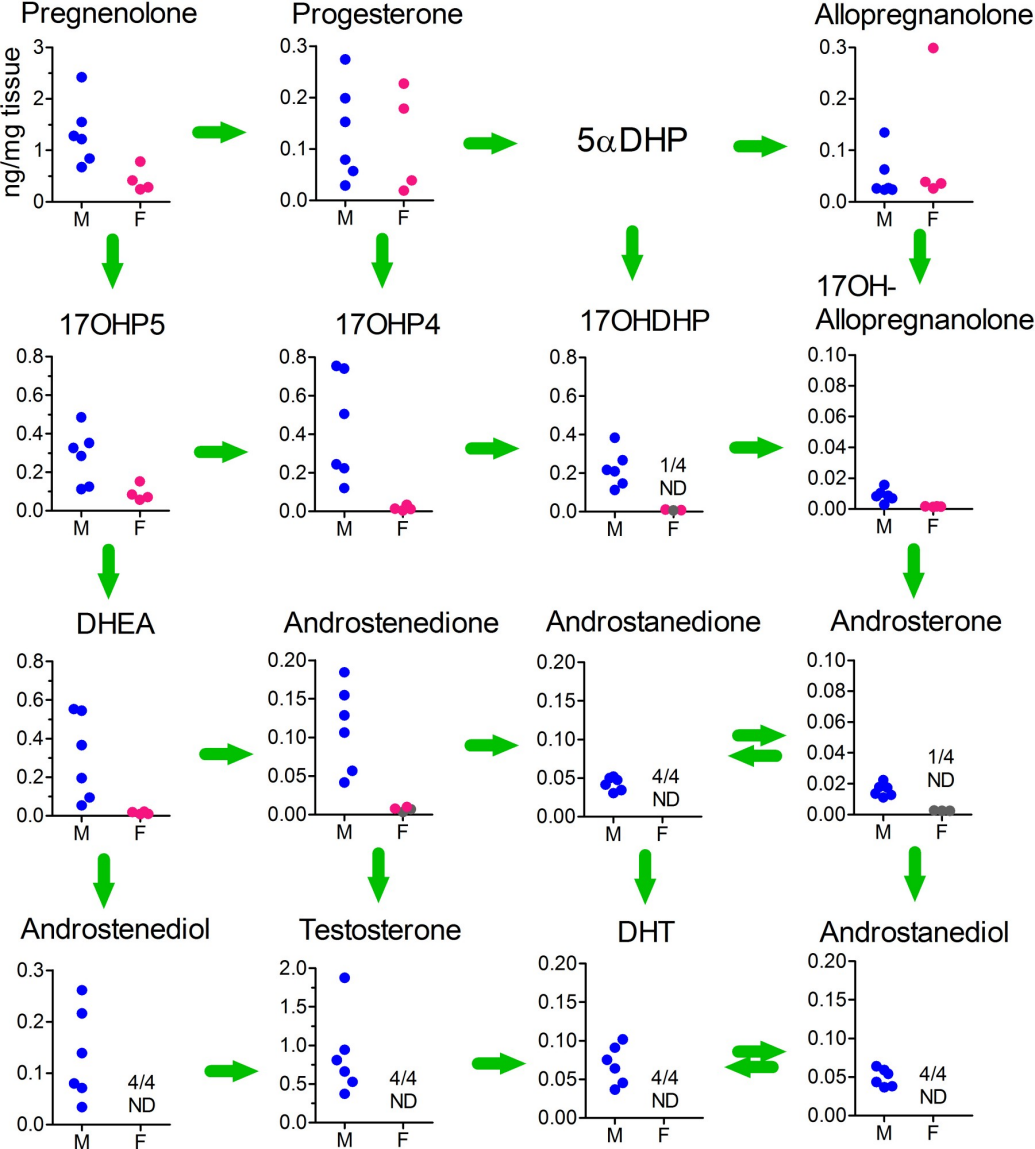
Levels of steroid intermediates involved in the canonical and backdoor synthesis of DHT in the testis and ovary as measured by GC-MS/MS. Intra-gonadal steroid levels from 6 male and 4 female fetuses (aged 15–19 weeks) are shown as individual points in each graph and arranged in the pathways shown in Fig 1. Levels of 5αDHP were not measured in this part of the study. The number of samples which were ND for each steroid are shown. Data shown in gray were above the LOD but below the formal LOQ, which means that the quantified data shown for these samples are less reliable. Raw data are shown in S1 Data (Sheet 3). androstanediol, 5α-androstan-3α, 17β-diol; androstanedione, 5α-androstane-3,17-dione; androstenediol, androst-5-ene-3β,17β-diol; DHEA, dehydroepiandrosterone; DHT, dihydrotestosterone; F, female; GC-MS/MS, gas chromatography–tandem mass spectrometry; LC-HRMS, liquid chromatography–high-resolution mass spectrometry; LOD, limit of detection; LOQ, limit of quantification; M, male; ND, nondetectable; 5αDHP, 5α-dihydroprogesterone; 17OHDHP, 17α-hydroxydihydroprogesterone.

To confirm the low/undetectable levels of 5α-reduced androgens in the fetal testis, testicular extracts from a further 6 fetuses were measured by GC-MS/MS to increase sensitivity (see Materials and methods), and 4 ovarian samples of a similar age (15–19 weeks) were included for comparison (Fig 4). In samples measured by GC-MS/MS, all of the backdoor steroids were detectable, but levels of 17α-hydroxyallopregnanolone and androsterone were very low. Low levels of androstanedione, androstanediol, and DHT were also detectable in all samples. Ovarian samples contained similar levels of progesterone and allopregnanolone compared with the testes, but all other steroids were reduced, and many were undetectable (Fig 4).

To determine whether human fetal testes produce backdoor steroids under hormonal stimulation, dispersed fetal testicular cells were incubated with or without human chorionic gonadotropin (hCG) for 24 hours, and the steroids produced were measured by GC-MS/MS. In the canonical pathway, pregnenolone, DHEA, and androstenedione were detectable in most samples, as was pregnenolone at low levels (S4 Fig). The presence of hCG had a stimulatory effect on DHEA levels. DHT was detected in one control culture. No backdoor androgens, or intermediates in their synthesis, were detectable in any testicular cell cultures.

### Enzymes associated with the backdoor pathway in the male fetus are predominantly expressed in nongonadal tissues

**Fig 5:**
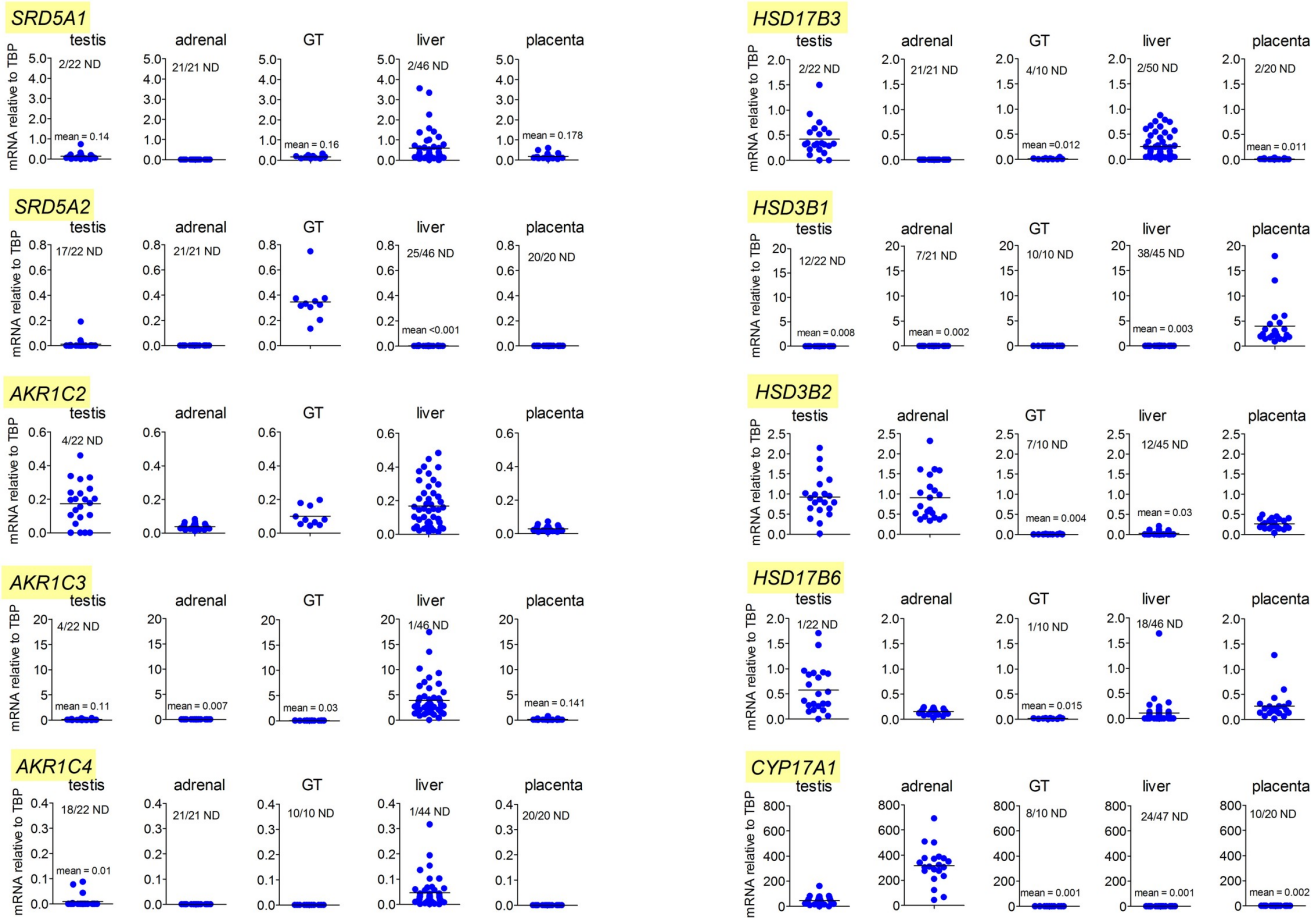
Levels of mRNA transcripts encoding enzymes involved in the synthesis of DHT through the backdoor pathway in the fetal male. Data show levels of transcripts in testis (*n* = 22), adrenal (*n* = 21), liver (*n* = 44–50), genital tubercle (*n* = 10), and placenta (*n* = 20) from individual male fetuses during the second trimester. Transcript levels have been measured relative to the housekeeping gene *TBP*. For each transcript, the y-axis has been maintained constant for all 5 tissues so that direct comparison of transcript levels can be made. The number of ND samples is shown on each graph. The horizontal black bar indicates mean expression and, in cases in which levels are very low, the mean is also provided in text on the graph. Raw data are shown in S1 Data (Sheet 4). DHT, dihydrotestosterone; GT, genital tubercle; ND, nondetectable; TBP, TATA box–binding protein.

The critical entry point to the backdoor pathway is through 5α-reduction of progesterone or 17α-hydroxyprogesterone by steroid 5α-reductase type 1 (SRD5A1). The highest levels of *SRD5A1* expression in the second trimester male fetus were in the liver, with significant but lower expression in the placenta, testis, and genital tubercle (Fig 5). Expression of *steroid 5α-reductase type 2* (*SRD5A2*) was only consistently detectable in the genital tubercle. The placenta and fetal liver are considerably larger than the other organs measured in this study (Table 1) and, in terms of total fetal transcript levels, therefore, these tissues have about 1,000 times greater *SRD5A1* expression than the testis. AKR1C2 is specific to the backdoor pathway and is critical for human fetal masculinization [15]. Mean *AKR1C2* transcript levels were highest in the fetal liver and fetal testis (Fig 5), although taking tissue mass into account, liver and placenta each have about 200 times more total *AKR1C2* transcript than the fetal testes. There was also significant *AKR1C2* expression in the genital tubercle, which is likely to be important for local DHT synthesis from androsterone (Fig 1). AKR1C4 is the other backdoor enzyme that may be required for masculinization [15], and transcripts were only consistently detected in the fetal liver (Fig 5). Expression of *17β-hydroxysteroid dehydrogenase type 6* (*HSD17B6*) was highest in the testis, with transcripts also consistently detected in the adrenal and placenta, and lower expression in the genital tubercle (Fig 5). Transcripts encoding 17β-hydroxysteroid dehydrogenase type 3 (HSD17B3) were expressed at similar levels in the fetal testis and liver, with very low or undetectable levels in the placenta, adrenal, and genital tubercle (Fig 5). Levels of *aldo-keto reductase type 1C3* (*AKR1C3*) transcript were highest in the liver, with low but detectable expression in other tissues. The cytochrome P450 enzyme 17α-hydroxylase/17,20 lyase (CYP17A1) is essential in both canonical and backdoor pathways of androgen synthesis (Fig 1). Predictably, expression was high in both fetal adrenal and fetal testis, with the mean adrenal level about 6 times that of the testis (Fig 5). Expression was very low or undetectable in the genital tubercle, liver, and placenta. The enzyme 3β-hydroxysteroid dehydrogenase (HSD3B) is essential for de novo androgen synthesis (in both canonical and backdoor pathways), with two isoforms (*HSD3B1* and *HSD3B2*) present on the human genome. Transcripts encoding HSD3B1, which has a 5- to 10-fold higher substrate affinity than HSD3B2 [23], were highly expressed in the placenta, with levels in other tissues either very low or undetectable. *HSD3B2* was expressed predominantly in the testis and adrenal, although the placenta also contained *HSD3B2* transcripts, and some low-level expression was seen in the fetal liver (Fig 5).

**Table 1:** Fetal tissue weights during the second trimester.

Tissue	Tissue weight (mg)*	Fold differences versus combined testis weights	References
Liver	700–14,800	117–220	our data and [24]
Testis (combined)	6–67	1–1	our data
Adrenal (combined)	50–1,300	8–19	our data
Genital tubercle	3–98	0.5–1.5	our data
Placenta	36,200–126,880	5,900–1,894	our data

*Range of tissue weights from 12–19 weeks of gestation in the human.

## Discussion

Masculinization of the fetus is dependent on the action of testosterone at the Wolffian ducts and on the action of DHT at the external genitalia [25]. The process of masculinization at the external genitalia starts in the late first/early second trimester, and the most intense phase of penile growth occurs later in the second trimester [8]. This is a critical period for normal masculinization, therefore, and it was assumed until recently that growth of the external genitalia was solely dependent on DHT formed in the target organ through 5α-reduction of testis-derived testosterone. However, the recent demonstration that the alternative, backdoor pathway to DHT synthesis is also required for normal human fetal male development [15,16] has shown that the process involves a complex interaction between different steroidogenic pathways. In this study, we now show that androsterone is the major circulating backdoor androgen in the human male fetus and that androsterone and testosterone are the only fetal androgens that are higher in the male circulation than the female circulation. In addition, most circulating androsterone in the fetal male appears to come from nongonadal tissues, probably using placental progesterone (or its metabolites) as substrate. Masculinization depends, therefore, not only on the fetal testes but also on other, nongonadal tissues.

Data in Fig 2 indicate that in the fetal human, the backdoor pathway of androgen synthesis appears to depend initially on progesterone formed from pregnenolone, and in the fetus, the major de novo sources of pregnenolone are the adrenal and the testis. In both tissues, however, pregnenolone is metabolized predominantly through the Δ^5^ pathway to DHEA because pregnenolone is bound with a significantly higher affinity by human CYP17A1 (Michaelis constant [Km] of 0.8 μM) than by HSD3B2 (Km of 5.5 μM) [26,27]. In addition, HSD3B2 activity is likely to be relatively low compared with CYP17A1 in both tissues, based on transcript levels (this study and [28–30]). It is likely, therefore, that most progesterone in the fetal circulation comes from the placenta, which secretes progesterone directly into the fetal circulation at high levels [31,32], similar to those reported here. Several other human fetal tissues express *CYP11A1* and may be capable of pregnenolone synthesis [30] but, based on transcript levels, they are likely to make a minor overall contribution to plasma levels.

Metabolism of progesterone through the backdoor pathway requires the enzymes SRD5A1, AKR1C2, and CYP17A1. Only the testes consistently express transcripts encoding all three enzymes, which would suggest that they are a likely source of backdoor androgens. Direct measurement of intratesticular steroid levels shows, however, that the testes contain only very low levels of androsterone and the potential substrate 17α-hydroxyallopregnanolone. Levels are lower than those of DHT and androstenedione, which are undetectable in fetal plasma (Fig 2), making it highly unlikely that testicular androsterone contributes significantly to fetal plasma levels. Enzyme kinetics suggest that in tissues such as the testes, which express all the necessary enzymes, the backdoor pathway can go from progesterone either through 17α-hydroxyprogesterone (CYP17A1 Km for progesterone 0.7 μM [26]) to 17α-hydroxyallopregnanolone or through 5αDHP (SRD5A1 Km for progesterone 0.8 μM [33]) and 17α-hydroxydihydroprogesterone (CYP17A1 Km for 5αDHP 0.2μM) to 17α-hydroxyallopregnanolone [19]. The failure of the testes to generate significant levels of 17α-hydroxyallopregnanolone may mean, therefore, that 17α-hydroxydihydroprogesterone is not a good substrate for human AKR1C2. The alternative pathway through allopregnanolone is also limited by a relatively low affinity between allopregnanolone and CYP17A1 (Km 18 μM), although 17α-hydroxyallopregnanolone is an excellent substrate for C17-20 lyase activity (Km 0.6 μM) [19].

In tissues that lack CYP17A1 but express SRD5A1 and AKR1C2, progesterone will be metabolized to allopregnanolone and potentially become available for metabolism by other tissues that express CYP17A1. Plasma steroid levels would support this pathway, although formation of 17α-hydroxyallopregnanolone is again limiting, probably reflecting the low affinity between allopregnanolone and CYP17A1. This pathway depends initially on SRD5A1, and transcript levels of this enzyme are highest in the liver (Fig 5), with lower levels also present in the placenta and testis, consistent with earlier studies of enzyme activity [34]. Tissue levels of 5αDHP are highest in the placenta, however, which probably reflects the high concentration of progesterone substrate in this tissue. The Km of SRD5A1 with progesterone as substrate (0.8 μM) [33] is equivalent to about 250 ng/mL or 0.25 ng/mg tissue (assuming a tissue density of 1). The mean progesterone concentrations in the placenta and liver are 6 ng/mg and 0.4 ng/mg, which means that, in most samples, they exceed the enzyme Km. The highest consistent tissue concentrations of allopregnanolone are found in the placenta and fetal liver (Fig 3). Both AKR1C2 and AKR1C4 have a Km of 0.6 μM with 5αDHP as substrate [35] (or 0.2 ng/mg tissue), which approaches 5αDHP concentrations in the liver (mean 0.07 ng/mg) and is exceeded by concentrations in the placenta (mean 0.3 ng/mg). High concentrations of allopregnanolone in the fetal liver are likely, therefore, to be a reflection of high AKR1C2 and AKR1C4. The placenta does not contain high levels of *AKR1C2* transcript, and *AKR1C4* is absent, but high substrate levels and lack of further metabolism via CYP17A1 probably explains the high tissue levels of allopregnanolone. The fetal adrenals do not express *SRD5A1/2* and would not be expected to contribute to fetal 5αDHP or allopregnanolone production. Taking tissue mass into account, the liver and placenta are likely, therefore, to be the major sites of 5αDHP and allopregnanolone production in the second trimester fetus. Placental allopregnanolone production would also be consistent with the increasing plasma levels in pregnant women during gestation [36].

Circulating levels of backdoor steroids suggest that, in the fetus, the pathway goes largely through allopregnanolone and 17α-hydroxyallopregnanolone to androsterone. Allopregnanolone levels in human fetal plasma are relatively high (male, about 200 ng/mL) but androsterone levels are significantly lower in comparison (male, about 3 ng/mL), which suggests that this is the limiting step in the pathway, probably reflecting the low affinity of human CYP17A1 for allopregnanolone. In female fetuses, allopregnanolone plasma levels are higher than in males, but downstream metabolites in the backdoor pathway are all reduced. This means that sex differences in the backdoor pathway are most likely to arise from reduced CYP17A1 activity in the female. *CYP17A1* is expressed in many fetal tissues [30] but, with the exception of the adrenal and testis, transcript levels are low ([30] and this study), suggesting enzyme activity is also probably low. In males, tissue androsterone levels are highest in the adrenals, which is consistent with high *CYP17A1* transcript levels and with a previous study showing androsterone present in the fetal adrenal at the end of the first trimester [28]. The substrate for this reaction must, however, come from circulating allopregnanolone, because the adrenals lack *SRD5A1* and appear unable to make 5α-reduced steroids. Our studies also show that the placenta contains detectable androsterone in most samples. There is some debate about whether the human placenta expresses significant CYP17A1 [37,38], but our data show that, in the samples used for this study, transcripts are either absent or are present only at very low levels. This suggests, therefore, that most placental androsterone is likely to be derived from adrenal DHEA. Given the relative sizes of the fetal organs involved and the tissue concentrations, the placenta and the fetal adrenals are likely to be the major sources of androsterone production in the male fetus. Many human tissues show sexual dimorphism, including the placenta, fetal liver, and fetal adrenal [21, 39, 40]. However, the fetal liver and placenta are unlikely to be responsible for sex differences in circulating androsterone, as they express little or no CYP17A1 and so will not metabolize allopregnanolone (the step at which sex differences are first seen in the backdoor pathway). In addition, we have measured androsterone levels in female fetal adrenals, and they do not differ significantly from the male (male 0.330 ng/mg, female 0.342 ng/mg; 7/30 nondetectable in the male group, 8/30 nondetectable in the female group. S1 Data, Sheet 2 and Sheet 6). Thus, while it remains to be established how sex differences in plasma androsterone levels arise, our data show that the testes are unlikely to be the primary cause and that differences in synthesis or metabolism must occur elsewhere.

Sex differences in plasma testosterone were expected from our current understanding of sexual development and from earlier studies on human fetal plasma and amniotic fluid levels [41–45]. Nevertheless, despite the clear sex difference in mean plasma testosterone levels, there was considerable overlap between individual samples from either sex. Similar overlaps in plasma levels has been reported in one previous study [44] but not another [45], while animal studies would suggest that overlapping testosterone levels in the fetus is common [46]. It is perhaps not surprising that testosterone is detectable in most female samples, as levels of both potential substrates (androstenedione and androstenediol) are present in the circulation, and the enzymes necessary to form testosterone (HSD3B and HSD17B3) are expressed in nongonadal tissues.

**Fig 6:**
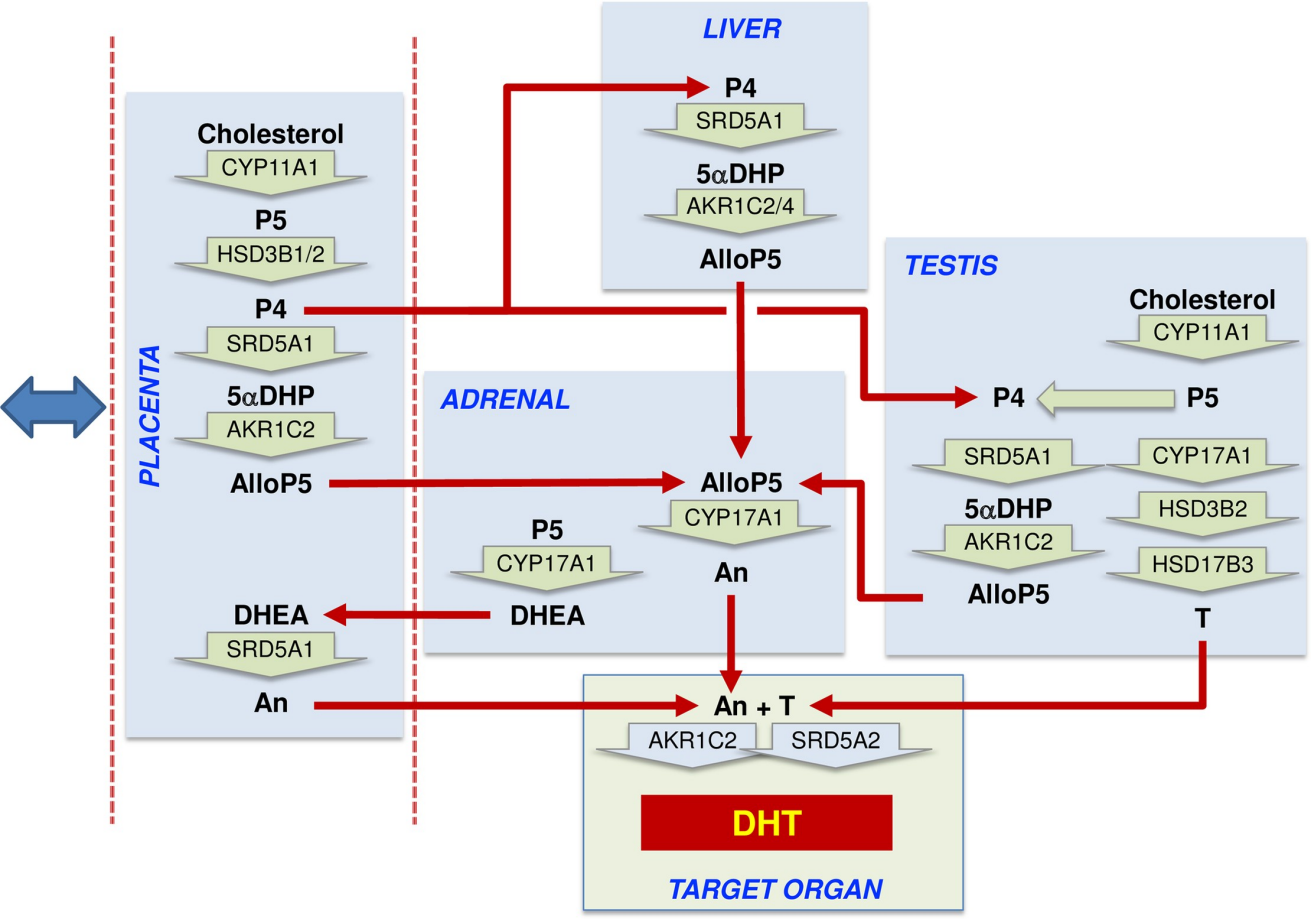
Proposed steroidogenic pathways leading to androsterone synthesis and masculinization in the second trimester human male fetus. Steroid hormone conversion is shown by wide green arrows, with the converting enzymes written within the arrow. Red arrows show potential transport between organs in the fetal circulation. The blue double-headed arrow indicates that exchange is also taking place between the placenta and the maternal circulation. Most circulating progesterone in the fetal circulation is likely to come from the placenta, and this will be reduced to 5αDHP by SRD5A1 in the placenta, fetal liver, and fetal testis, with the fetal liver likely to be the major site. Allopregnanolone (AlloP5) production by AKR1C2 is also most likely to occur in the placenta and fetal liver because the substrate is present in those tissues, and they express the highest total levels of enzyme transcript. Some conversion may also occur in the testis. Significant levels of androsterone are only detectable in the placenta and adrenal, and thus they are a likely source of the circulating steroid, although, given sex differences, other tissues are probably involved. The adrenal lacks other intermediates in the backdoor pathway, and thus AlloP5 must come from other tissues. The placenta lacks *CYP17A1*, so androsterone production is likely to depend on adrenal DHEA as substrate. Testosterone from the fetal testes also acts as an essential substrate for DHT synthesis at the external genitalia. AlloP5, allopregnanolone; An, androsterone; DHEA, dehydroepiandrosterone; DHT, 5α-dihydrotestosterone; P4, progesterone; P5, pregnenolone; T, testosterone; 5αDHP, 5α-dihydroprogesterone.

When the backdoor pathway was shown to be essential for human fetal masculinization, it was suggested that DHT is synthesized via this pathway in the fetal testes and released into the circulation [15,16]. We now show that this is very unlikely because DHT is undetectable, or present at very low levels, in testes from most fetuses and because circulating DHT levels are undetectable (<1 ng/mL). These results are consistent with earlier studies that either failed to detect DHT in the human fetal testis [47,48] or found very low levels [49]. DHT can be metabolized to androstanediol by all AKR1 enzymes, and AKR1C2 in particular [12,50], and given the high levels of AKR1 enzyme transcripts in the fetal liver, it is likely that any DHT released into the fetal circulation is rapidly metabolized. Our results show, therefore, that the major circulating backdoor androgen in the fetus is androsterone, which is present at similar levels to testosterone and is significantly higher in male plasma than female plasma. It remains to be shown whether the genital tubercle can convert androsterone to DHT, but the tissue expresses high levels of *AKR1C2*, as well as detectable *AKR1C3*, and AKR1C2 can catalyze both oxidation and reduction steps required for androsterone conversion to DHT (Figs 1 and 5). A schematic diagram of the proposed pathways involved in backdoor androgen synthesis in the human fetal male is shown in Fig 6.

In the DSD cases described by Flück and colleagues [15], the 46,XY karyotype patients carried hypomorphic mutations in either *AKR1C2* and *AKR1C4*, or *AKR1C2* alone. From data reported here, reduced fetal AKR1C2 activity would be expected to affect backdoor pathways in the liver and testis, reducing production of allopregnanolone. In addition, loss of enzyme activity in the genital tubercle may affect production of DHT at the target organ. The potential involvement of placental AKR1C2 in the reported 46,XY DSD patients is also of interest. The placenta develops from both maternal and fetal cells, and it is not known whether placental AKR1C2 is of fetal or maternal origin. It is of note, however, that two 46,XY individuals who are known heterozygotes for a mutation in *AKR1C2* show divergent phenotypes, with one showing a normal male phenotype and the other DSD [15]. The mother of the affected individual is also heterozygous for a mutation in *AKR1C2*, and if placental AKR1C2 is of maternal origin, then this would be expected to affect backdoor steroid production in the placenta. Unfortunately, the genotype of the mother of the unaffected heterozygous individual is not available.

Further strong evidence for a nontesticular pathway of backdoor androgen synthesis comes from patients with P450 oxidoreductase (POR) or 21-hydroxylase deficiency who show 46,XX virilization. It is likely that virilization in utero in individuals with 21-hydroxylase deficiency is due to excessive backdoor androgen synthesis that is occurring despite the absence of testes [51]. In individuals with POR deficiency, the effects are more complex, as POR supports many P450 enzymes, and specific POR mutations can affect different enzymes [52–54]. Virilization of 46,XX individuals can occur, for example, because of reduced placental CYP19A1 activity leading to accumulation of placental androgens [55]. It has also been shown, however, that some mutations of POR that have less effect on aromatase are associated with increased backdoor androgen production [56,57]. It has been postulated that the increase in fetal backdoor androgen production seen in POR or 21-hydrdoxylase deficiency comes from the fetal adrenals [58,59]. However, the lack of SRD5A1 and intermediates in the backdoor pathway in normal fetal adrenals make this unlikely, unless the condition itself increases adrenal SRD5A1 activity (e.g., through increased adrenal stimulation by adrenocorticotropic hormone). Our data would suggest that the increased fetal adrenal 17α-hydroxyprogesterone seen in these conditions acts initially as substrate for backdoor androgen production through 5α-reduction in other tissues—probably the fetal liver.

If the placenta is a critical component of the fetal backdoor androgen pathway, as suggested by these data, then it has implications for our understanding of the regulation of masculinization and DSD. It is now established that placental insufficiency, associated with intrauterine growth restriction (IUGR), is associated with abnormalities in development of the male external genitalia, and hypospadias in particular [60,61]. Severe forms of placenta-mediated IUGR start during the first trimester [60] and could interfere, therefore, with all aspects of fetal masculinization. It has been suggested that DSD associated with placental dysfunction could be due to reduced placental hCG production [61], but other studies have shown that maternal hCG levels tend to be increased in placental insufficiency [60]. In contrast, maternal progesterone levels are reported to be reduced during IUGR [62], suggesting that placental steroidogenesis is affected. If the placenta is central to fetal backdoor androgen production, as we suggest, then altered placental steroidogenesis may lead directly to abnormalities in masculinization.

Studies in the 1950s and 1960s by Jost and others showed that androgen is required for masculinization of the external genitalia (reviewed in [63]). Later studies found that testicular testosterone must be converted to DHT at the target organ to induce masculinization [64]. Most recently, it has been shown that the normal process of masculinization in the human depends on two separate pathways of androgen synthesis, the canonical and backdoor pathways [15]. We now report that the backdoor pathway in the human fetus is sexually dimorphic, even though the testes are unlikely to contribute significantly to the pathway. Instead, results suggest that the pathway is driven by placental progesterone production, which acts as substrate for androsterone synthesis, primarily in nongonadal tissues. Overall, these data show that current understanding of the endocrine control of masculinization in the human fetus should be revised. Our results indicate that the endocrine control of masculinization in the human fetus is mediated through circulating testosterone and androsterone and is dependent on a complex interaction and exchange between the testes and nongonadal tissues, particularly the placenta.

## Materials and methods

### Ethics statement

Collections of fetal material in Aberdeen and Stockholm and by the Human Developmental Biology Resource (HBDR) were respectively approved by the NHS Grampian Research Ethics Committees (REC 04/S0802/21 and REC 15/NS/0123), the Regional Ethics Committee of Stockholm (EPN dnr 2014/1022-32), and the relevant research ethics committees in accordance with the United Kingdom Human Tissue Authority (HTA; www.hta.gov.uk) Codes of Practice. Written, informed, maternal consent was received from participants prior to inclusion in the study.

### Sample collection

Three sources of human fetal tissues were used in this study: (1) in Aberdeen, human fetuses between 11 and 21 weeks of gestation and classified as normal at scan were collected from women over 16 years of age undergoing elective termination [21]. Fetal age was estimated initially by ultrasound scan, adjusted for days between scan and termination, and then cross-referenced with foot length. Information about maternal smoking during pregnancy was available for most fetuses. Fetuses were transported to the laboratory within 30 minutes of delivery, weighed, crown-rump length recorded, and sexed. Blood samples from a total of 42 male fetuses and 16 female fetuses were collected by cardiac puncture ex vivo, and plasma was stored at −80°C. Tissues were snap-frozen in liquid N_2_ and then stored at −80°C. (2) In Stockholm, human fetal testes were obtained for in vitro incubation studies after elective termination of pregnancy at 10–12 weeks of gestation. (3) Additional fetal livers and placentas were provided by the MRC/Wellcome-Trust–funded HBDR (http://www.hdbr.org). Available fetal and maternal characteristics relevant to samples used in different parts of this study are shown in S3 Table.

### RNA extraction, reverse transcription and real-time PCR

Total RNA was extracted from frozen fetal tissues either using TRIzol (Life Technologies, Paisley, UK) [65] or using Qiagen AllPrep kits (Qiagen, Manchester, UK). Reverse transcription, primer design, and real-time PCR were carried out as previously described [66,67], and the primers used are shown in S4 Table. RNA that is free of genomic DNA contamination is required to amplify *SRD5A1* because of the presence of a processed pseudogene in the genome, and this was carried out using RNAeasy Plus Micro-columns (Qiagen, Manchester, UK) followed by DNase treatment (DNA-free, Life Technologies, Paisley, UK). Transcripts encoding HSD3B1 and HSD3B2 have very similar sequences, but primers were designed that are specific to each transcript under the conditions used here (S4 Table). To normalize data, Normfinder was used to identify the most stable housekeeping genes in each tissue using the housekeeping genes and primers described earlier [68]. The best combinations of housekeeping genes varied between tissues and, thus, to allow comparison of transcript expression between tissues, TATA box–binding protein (*TBP*) was used as housekeeping gene for all samples, because it was the most consistently stable transcript across all tissues [68,69]. Some steroid enzyme transcript data (*SRD5A1*, *SRD5A2*, and *CYP17A1* in liver [69] and *HSD17B3* and *CYP17A1* in testis [70]) have been reported previously, relative to different housekeeping genes or external standards. These data are shown again here, relative to *TBP*, to allow comparisons between tissues. Additional fetal liver samples have also been included in the reported data. Fetal sex was confirmed by PCR of *ZFX* and *SRY* using genomic DNA and primers described in S4 Table [71].

### Isolation and incubation of human fetal testicular cells

Isolated fetal testes were treated with collagenase type I (Sigma, St. Louis, MO) (1 mg/mL for 35 minutes at 37°C) and then disrupted mechanically. The testicular cells were collected by centrifugation at 300*g* for 7 minutes, washed in Hank’s balanced salt solution, and resuspended in DMEM-F12 supplemented with 1 mg/mL BSA, 100 IU/mL penicillin and 100 μg/mL streptomycin. For incubation, 100 μL of a suspension containing 1.5 × 10^5^ cells/mL was plated onto 96-well plates (Falcon, Franklin Lakes, NJ) in the presence or absence of hCG (10 ng/mL) to stimulate Leydig cell activity. Cells were incubated for 24 hours at 37°C under 5% CO_2_, and steroid levels in the culture media were measured by GC-MS/MS as described below.

### Steroid extraction and quantification

Methods used to extract and profile steroid levels by GC-MS/MS in fetal plasma and culture media have been described elsewhere in detail [28,72,73]. Briefly, fetal plasma samples (50 μL) or culture media samples (200 μL) were spiked with internal standards (stable isotope-labeled steroid analogues) and an enzymatic deconjugation of phase II metabolites was performed (Sulfatase, Sigma S9626 [100 U/mL] and β-Glucuronidase, Sigma G8132 [5 KU/mL]). Steroids were extracted twice with diethyl ether and a ChromP SPE cartridge was used for initial purification. Androgens and estrogens were separated by liquid/liquid partitioning with *n*-pentane and were further purified on a silica SPE cartridge. The androgen fraction was derivatized with MSTFA/TMIS/DTE, and 2 μL of each extract was injected onto a Scion 436 gas chromatograph coupled to a Scion TQ triple quadrupole mass spectrometer (Bruker, Fremont, CA). Electron ionization (70 eV) was used and two diagnostic signals (SRM acquisition mode) were monitored for identification and quantification of the targeted compounds. Levels of circulating testosterone in male fetuses, shown in detail here, have been reported previously as mean levels [74].

Extraction and quantification of tissue steroid levels by LC-HRMS have been described previously [21]. Tissue samples (25–65 mg) from placenta, liver, and adrenals or whole testes (8–20 mg, one per fetus) were initially processed to isolate RNA/DNA/protein using Qiagen Allprep kits, as above, and steroids were then extracted from the column-eluants following addition of internal standards [21]. Following extraction, steroids were separated by reverse-phase liquid chromatography on a PFP column with trimethyl silane (TMS) endcapping (50 mm, 2.1 mm, 2.6 μm, Accucore, Thermo Fisher Scientific, Waltham, MA) and using an UltiMate 3000 RSLCnano autosampler/pump (Thermo Fisher Scientific). Steroids were then ionized by electrospray in positive mode, and signals were recorded on a Q Exactive Orbitrap (Thermo Fisher Scientific) mass spectrometer. Sulfated steroids were detected in a second analysis after negative electrospray ionization. Levels of sulfated and nonsulfated DHEA have been combined in the reported results, as have sulfated and nonsulfated androsterone. The fetal testes used in this part of the study were extracted in two batches, and recovery of steroid sulfates in the first batch was poor, so testicular DHEA and androsterone levels have only been reported for the second batch of 10 samples. For the second batch of testicular tissue, an additional extraction step using chloroform/*n*-butanol was used to improve steroid sulfate extraction efficiency. As reported for other LC-MS methods [75], the electrospray ionization efficiency can be low for 5α-reduced androgens, which results in a relatively high limit of quantification (LOQ) and limit of detection (LOD) value for these compounds. In a separate study, therefore, gonadal steroids from an additional 10 fetuses (6 male, 4 female) were extracted, and separation and quantification of steroids in these samples was carried out by GC-MS/MS as described above for plasma samples.

### Statistics

Data were checked for normality and heterogeneity of variance and normalized by log transformation as appropriate. Plasma steroid data were analyzed by two-factor ANOVA, with fetal age and maternal smoking as the factors. Correlations between steroid data and age were analyzed using Pearson correlation coefficient. The effect of hCG on steroid secretion by the fetal testes was analyzed by *t* test. Differences between male and female fetal plasma steroid levels were determined by *t* tests when 40% or more of the samples in each group had detectable levels of the steroid. The Cohen method of maximum likelihood estimation of the mean and variance was used to account for nondetectable samples in the analysis [76]. All raw data are shown in 5 separate sheets in S1 Data.

## Supporting information

S1 Fig Levels of epiandrosterone and etiocholanolone in male and female human fetal plasma during the second trimester. DHEA, androstenedione, and testosterone can be metabolized to epiandrosterone, and etiocholanolone and metabolite levels are shown here from the same samples as in Fig 2. Data shown in gray were above the LOD but below the formal LOQ, which means that the quantified data shown for these samples are less reliable. The red dotted line indicates the LOD. The *P*-value for etiocholanolone was calculated without including the outlier. Epiandrosterone is 5α-androstane-3β-ol-17-one, while etiocholanolone is 5β-androstan-3α-ol-17-one. Raw data are shown in S1 Data (Sheet 1). DHEA, dehydroepiandrosterone; F, female; LOD, limit of detection; LOQ, limit of quantification; M, male; ND, not detectable.
(TIF)

S2 Fig Age-dependent changes in human fetal male plasma steroid levels and effects of maternal smoking. Data are the same as those in Fig 2 but grouped according to fetal age and maternal smoking. Data points in black are from fetuses exposed to maternal smoking, while points in green are from nonexposed samples. Significant age-dependent effects were seen only with androstenediol and testosterone. Note that the androstenediol analysis was carried out without inclusion of the marked outlier at 16 weeks of gestation. There was no effect of maternal smoking on any of the steroids measured. The LOD, where appropriate, is shown by the horizontal broken line. Nondetectable data are excluded. Raw data are shown in S1 Data (Sheet 1). LOD, limit of detection.
(TIF)

S3 Fig Age-dependent changes in human fetal female plasma steroid levels and effects of maternal smoking. Data are the same as those in Fig 2 but grouped according to fetal age and maternal smoking. Data points in black are from fetuses exposed to maternal smoking, while points in green are from nonexposed samples. No significant age-dependent effects were seen, although there was a significant interaction between age and smoking for DHEA and androstenedione. Progesterone levels were significantly increased by maternal smoking. The LOD, where appropriate, is shown by the horizontal broken line. Nondetectable data are excluded. Raw data are shown in S1 Data (Sheet 1). DHEA, dehydroepiandrosterone; LOD, limit of detection.
(TIF)

S4 Fig Production of canonical and backdoor androgens by human fetal testicular cells in vitro. Cells were isolated from second trimester human fetal testes and incubated for 24 hours in the absence (green) or presence (blue) of hCG. Secreted steroids were measured by GC-MS/MS. The number of samples that were ND for each steroid are shown, and the limit of detection for each steroid is shown as a red dotted line. None of the steroids in the backdoor pathway were detectable. The data are derived from 4 fetuses (10–12 weeks), incubated on separate occasions. The effect of hCG on DHEA secretion was significant (*P* = 0.027). ND data are excluded. Raw data are shown in S1 Data (Sheet 5). DHEA, dehydroepiandrosterone; GC-MS/MS, gas chromatography–tandem mass spectrometry; hCG, human chorionic gonadotropin; ND, nondetectable.
(TIF)

S1 Table Table showing all steroids measured in human male fetal plasma by GC-MS/MS. Mean plasma levels (±SD) are shown; ND values were assigned a value of 50% of the LOD for illustration only. Mean values are only reported when 10 or more samples (about 25%) were detectable. GC-MS/MS, gas chromatography–tandem mass spectrometry; LOD, limit of detection; ND, nondetectable.
(DOCX)

S2 Table Table showing all steroids measured in human female fetal plasma by GC-MS/MS. Mean plasma levels (±SD) are shown; ND values were assigned a value of 50% of the LOD for illustration only. Mean values are only reported when 4 or more samples (25%) were detectable. GC-MS/MS, gas chromatography–tandem mass spectrometry; LOD, limit of detection; ND, nondetectable.
(DOCX)

S3 Table Fetal and maternal characteristics associated with samples used in this study. Fetuses were either from the Aberdeen FEGO study or, in the case of placenta and liver pairs, from the HDBR. Values are shown as mean ± SEM. Maternal smoking was not associated with any significant differences in the measured fetal or maternal characteristics. FEGO, Fetal Gonad; HDBR, Human Developmental Biology Resource.
(DOCX)

S4 Table List of all primers used for qPCR and for sexing fetuses. qPCR, quantitative PCR.
(DOCX)

S1 Data Lists of all numerical data reported in this study. (Sheet 1) Plasma steroid levels in male and female fetuses. (Sheet 2) Tissue steroid levels measured by LC-MS/MS. (Sheet 3) Steroid levels in fetal gonads measured by GC-MS/MS. (Sheet 4) Transcript levels measured by qPCR relative to the housekeeping gene (TBP). (Sheet 5) Steroids measured in cell culture studies. (Sheet 6) Androsterone sulfate levels in female fetal adrenals. GC-MS/MS, gas chromatography–tandem mass spectrometry; LC-MS/MS, liquid chromatography-high resolution mass spectrometry; qPCR, quantitative PCR; TBP, TATA box–binding protein.
(XLSX)

## Abbreviations

AKR1C2: aldo-keto reductase type 1C2
AKR1C3: aldo-keto reductase type 1C3
AKR1C4: aldo-keto reductase type 1C4
androstanediol: 5α-androstan-3α,17β-diol
CYP17A1: cytochrome P450 type 17A1
DHEA: dehydroepiandrosterone
DHT: dihydrotestosterone
DSD: disordered sex development
GC-MS/MS: gas chromatography–tandem mass spectrometry
HBDR: Human Developmental Biology Resource
hCG: human chorionic gonadotropin
HSD17B6: 17β-hydroxysteroid dehydrogenase type 6
HSD3B: 3β-hydroxysteroid dehydrogenase
HTA: Human Tissue Authority
IUGR: intrauterine growth restriction
Km: Michaelis constant
LC-HRMS: liquid chromatography–high-resolution mass spectrometry
LOD: limit of detection
LOQ: limit of quantification
POR: P450 oxidoreductase
qPCR: quantitative PCR
SRD5A1: steroid 5α-reductase type 1
SRD5A2: steroid 5α-reductase type 2
SULT2A1: sulfotransferase type 2A1
TBP: TATA box–binding protein
TMS: trimethyl silane
5αDHP: 5α-dihydroprogesterone
