# Supplementary figures and images for: Alternative (backdoor) androgen production and masculinization in the human fetus
Source: PLoS Biol. 2019 Feb 14;17(2):e3000002. doi: 10.1371/journal.pbio.3000002 (PMC6375548; doi:10.1371/journal.pbio.3000002)

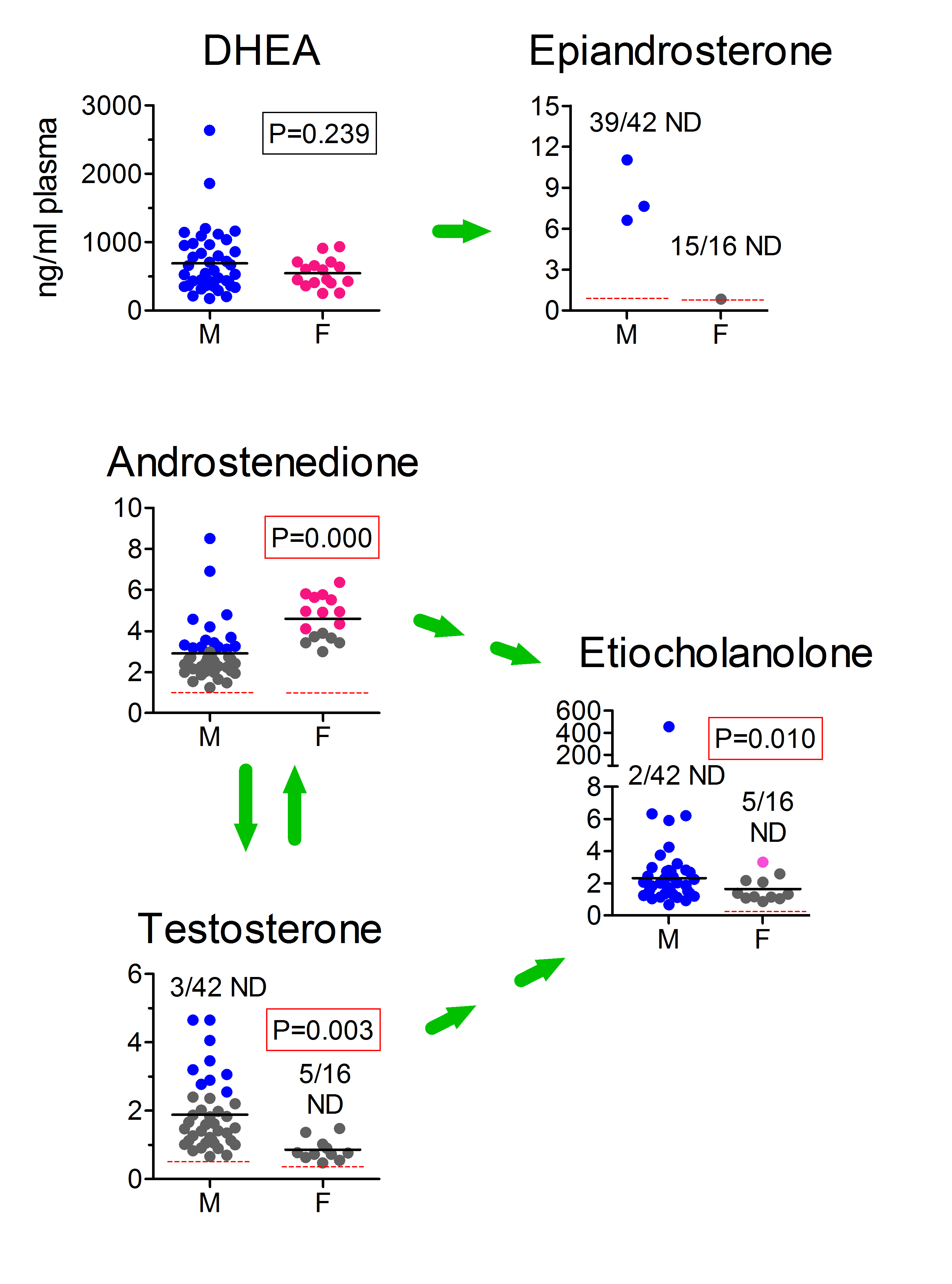

Supplement: S1 Fig — DHEA, androstenedione, and testosterone can be metabolized to epiandrosterone, and etiocholanolone and metabolite levels are shown here from the same samples as in Fig 2. Data shown in gray were above the LOD but below the formal LOQ, which means that the quantified data shown for these samples are less reliable. The red dotted line indicates the LOD. The P-value for etiocholanolone was calculated without including the outlier. Epiandrosterone is 5α-androstane-3β-ol-17-one, while etiocholanolone is 5β-androstan-3α-ol-17-one. Raw data are shown in S1 Data (Sheet 1). DHEA, dehydroepiandrosterone; F, female; LOD, limit of detection; LOQ, limit of quantification; M, male; ND, not detectable. (TIF) [file pbio.3000002.s001.tif]

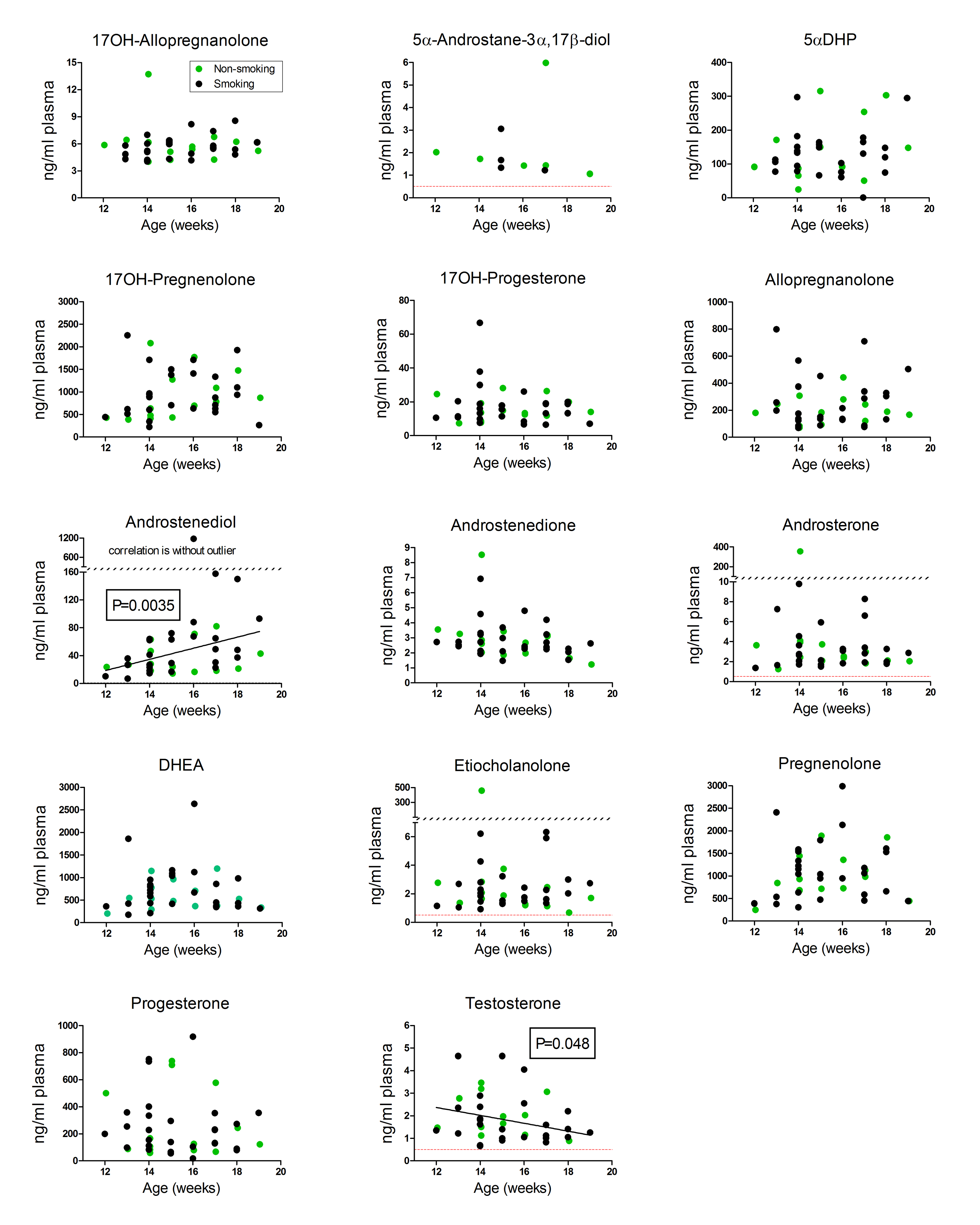

Supplement: S2 Fig — Data are the same as those in Fig 2 but grouped according to fetal age and maternal smoking. Data points in black are from fetuses exposed to maternal smoking, while points in green are from nonexposed samples. Significant age-dependent effects were seen only with androstenediol and testosterone. Note that the androstenediol analysis was carried out without inclusion of the marked outlier at 16 weeks of gestation. There was no effect of maternal smoking on any of the steroids measured. The LOD, where appropriate, is shown by the horizontal broken line. Nondetectable data are excluded. Raw data are shown in S1 Data (Sheet 1). LOD, limit of detection. (TIF) [file pbio.3000002.s002.tif]

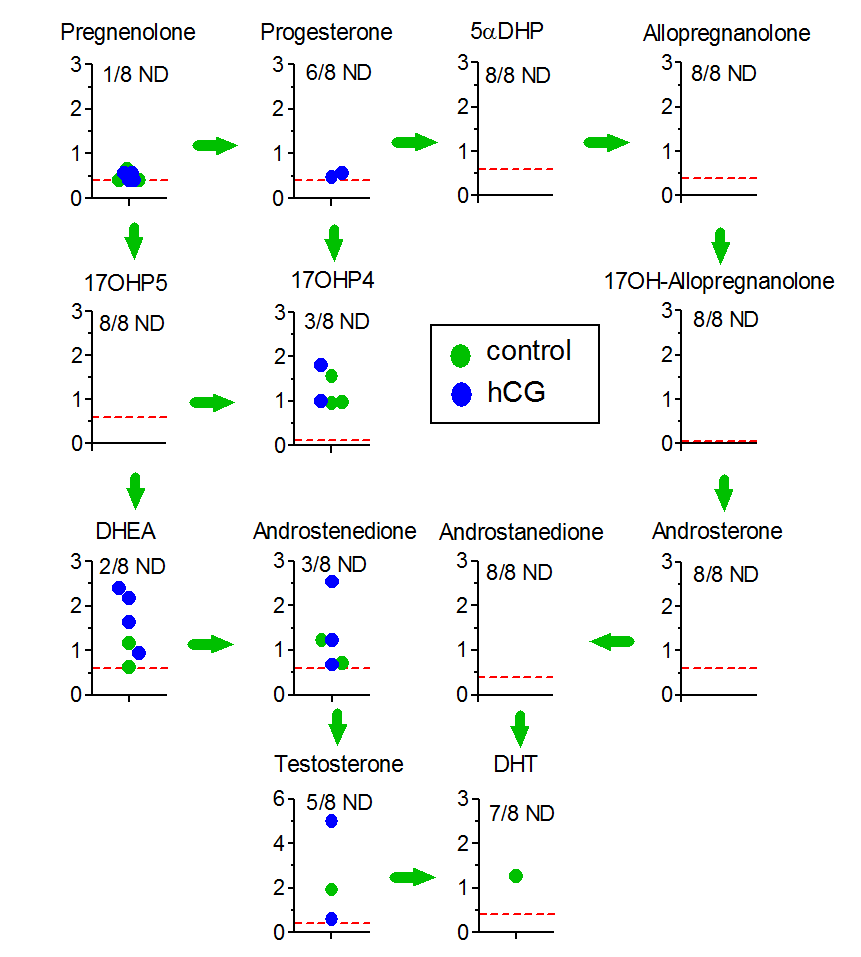

Supplement: S4 Fig — Cells were isolated from second trimester human fetal testes and incubated for 24 hours in the absence (green) or presence (blue) of hCG. Secreted steroids were measured by GC-MS/MS. The number of samples that were ND for each steroid are shown, and the limit of detection for each steroid is shown as a red dotted line. None of the steroids in the backdoor pathway were detectable. The data are derived from 4 fetuses (10–12 weeks), incubated on separate occasions. The effect of hCG on DHEA secretion was significant (P = 0.027). ND data are excluded. Raw data are shown in S1 Data (Sheet 5). DHEA, dehydroepiandrosterone; GC-MS/MS, gas chromatography–tandem mass spectrometry; hCG, human chorionic gonadotropin; ND, nondetectable. (TIF) [file pbio.3000002.s004.tif]
