# Supplementary material for: Alternative (backdoor) androgen production and masculinization in the human fetus
Source: PLoS Biol. 2019 Feb 14;17(2):e3000002. doi: 10.1371/journal.pbio.3000002 (PMC6375548; doi:10.1371/journal.pbio.3000002)
Supplement: S1 Table — Mean plasma levels (±SD) are shown; ND values were assigned a value of 50% of the LOD for illustration only. Mean values are only reported when 10 or more samples (about 25%) were detectable. GC-MS/MS, gas chromatography–tandem mass spectrometry; LOD, limit of detection; ND, nondetectable. (DOCX) [file pbio.3000002.s005.docx]

Systematic name trivial name detected LOD (ng/ml) Mean ± sd (ng/ml)

5α-pregnan-3α-ol-20-one allopregnanolone 38/38 0.2 243 ± 174

5α-pregnan-3α,17α-diol-20-one 17α-hydroxyallopregnanolone 38/38 0.2 5.76 ± 1.71

5α-pregnane-3,20-dione dihydroprogesterone 37/38 1 135 ± 75.12

5α-pregnan-17α-ol-3,20-dione 17α-hydroxydihydroprogesterone 0/38 1

pregn-5-ene-3β-ol-20-one pregnenolone 42/42 0.5 1066 ± 612

pregn-5-en-17α,3β-diol-20-one 17α-hydroxypregnenolone 42/42 0.5 962 ± 528

pregn-4-ene-3,20-dione progesterone 42/42 0.5 258 ± 229

17α-hydroxypregn-4-ene-3,20-dione 17α-hydroxyprogesterone 42/42 0.5 16.8 ± 10.5

5α-androstane-3,17-dione androstanedione 0/38 1

5β-androstane-3,17-dione 5β-androstanedione 0/38 1

5β-androstan-3α, 17α-diol 0/42 5

5β-androstan-3α, 17β-diol 5β-androstanediol 1/41 5

5β-androstane-3β, 17α-diol 0/42 1

5β-androstane-3β, 17β-diol 0/42 1

5α-androstan-3α, 17α-diol 0/42 1

5α-androstan-3α, 17β-diol androstanediol 10/42 1 0.878 ± 0.974

5α-androstane-3β, 17α-diol 0/42 1

5α-androstane-3β, 17β-diol 0/42 1

5β-androstane-17β-ol-3-one 5β-dihydrotestosterone 0/42 1

5α-androstan-3α-ol-17-one androsterone 41/42 1 11.4 ± 54.1

5α-androstane-3β-ol- 17-one epiandrosterone 3/42 1

5β-androstan-3α-ol-17-one etiocholanolone 40/42 1 13.1 ± 70.4

5α-androstane-17β-ol-3-one dihydrotestosterone 0/42 1

Androst-5-en-3α,17β- diol 0/42 1

androst-5-ene-3β,17β-diol androstenediol 42/42 1 71.8 ± 178

androst-5-ene-3β,17α- diol 1/42 1

androst-5-ene -3β-ol-17-one dehydroepiandrosterone 42/42 1 692 ± 468

androst-4-ene -3,17-dione androstenedione 42/42 1 2.90 ± 1.35

androst-4-en -17α-ol-3-one epitestosterone 0/42 0.5

androst-4-ene -17β-ol-3-one testosterone 39/42 0.5 1.77 ± 1.09
