# Supplementary material for: Alternative (backdoor) androgen production and masculinization in the human fetus
Source: PLoS Biol. 2019 Feb 14;17(2):e3000002. doi: 10.1371/journal.pbio.3000002 (PMC6375548; doi:10.1371/journal.pbio.3000002)
Supplement: S2 Table — Mean plasma levels (±SD) are shown; ND values were assigned a value of 50% of the LOD for illustration only. Mean values are only reported when 4 or more samples (25%) were detectable. GC-MS/MS, gas chromatography–tandem mass spectrometry; LOD, limit of detection; ND, nondetectable. (DOCX) [file pbio.3000002.s006.docx]

Systematic name trivial name detected LOD (ng/ml) Mean ± sd (ng/ml)

5α-pregnan-3α-ol-20-one allopregnanolone 16/16 0.2 418 ± 340

5α-pregnan-3α,17α-diol-20-one 17α-hydroxyallopregnanolone 9/16 0.2 1.20 ± 1.14

5α-pregnan-17α-ol-3,20-dione 17α-hydroxydihydroprogesterone 6/16 1 2.13 ±3.03

pregn-5-ene-3β-ol-20-one pregnenolone 16/16 0.5 807 ± 274

pregn-5-en-17α,3β-diol-20-one 17α-hydroxypregnenolone 16/16 0.5 1023± 452

pregn-4-ene-3,20-dione progesterone 16/16 0.5 166 ± 95

17α-hydroxypregn-4-ene-3,20-dione 17α-hydroxyprogesterone 16/16 0.5 29.8 ± 12.4

5α-androstane-3,17-dione androstanedione 0/16 1

5β-androstane-3,17-dione 5β-androstanedione 0/16 1

5β-androstan-3α, 17α-diol 0/16 5

5β-androstan-3α, 17β-diol 5β-androstanediol 0/16 5

5β-androstane-3β, 17α-diol 0/16 1

5β-androstane-3β, 17β-diol 0/16 1

5α-androstan-3α, 17α-diol 0/16 1

5α-androstan-3α, 17β-diol androstanediol 0/16 1

5α-androstane-3β, 17α-diol 0/16 1

5α-androstane-3β, 17β-diol 0/16 1

5β-androstane-17β-ol-3-one 5β-dihydrotestosterone 0/16 1

5α-androstan-3α-ol-17-one androsterone 12/16 0.8 1.82 ± 2.01

5α-androstane-3β-ol- 17-one epiandrosterone 1/16 0.8

5β-androstan-3α-ol-17-one etiocholanolone 5/16 0.8 1.26 ± 0.88

5α-androstane-17β-ol-3-one dihydrotestosterone 0/16 1

Androst-5-en-3α,17β- diol 0/16 1

androst-5-ene-3β,17β-diol androstenediol 16/16 1 65.1 ± 56.7

androst-5-ene-3β,17α- diol 1/16 1

androst-5-ene -3β-ol-17-one dehydroepiandrosterone 16/16 1 547 ± 207

androst-4-ene -3,17-dione androstenedione 16/16 1 4.59 ± 1.04

androst-4-en -17α-ol-3-one epitestosterone 0/16 0.5

androst-4-ene -17β-ol-3-one testosterone 11/16 0.4 0.65 ± 0.41
