# Supplementary material for: Alternative (backdoor) androgen production and masculinization in the human fetus
Source: PLoS Biol. 2019 Feb 14;17(2):e3000002. doi: 10.1371/journal.pbio.3000002 (PMC6375548; doi:10.1371/journal.pbio.3000002)
Supplement: S3 Table — Fetuses were either from the Aberdeen FEGO study or, in the case of placenta and liver pairs, from the HDBR. Values are shown as mean ± SEM. Maternal smoking was not associated with any significant differences in the measured fetal or maternal characteristics. FEGO, Fetal Gonad; HDBR, Human Developmental Biology Resource. (DOCX) [file pbio.3000002.s007.docx]

Fetal and maternal characteristics. Fetuses were either from the Aberdeen FEGO study or, in the case of placenta and liver pairs, from the HDBR. Values are shown as mean±s.e.m. Maternal smoking was not associated with any significant differences in the measured fetal or maternal characteristics.

| **Fetuses used for plasma steroid hormone determination** | | | |
| --- | --- | --- | --- |
| Characteristics | | Male n=42 | Female n=16 |
| Fetal | Weeks of gestation | 15.3±0.3 | 16.0±0.4 |
|  | Body weight (g) | 97.1±10.0 | 112.7±16.0 |
|  | Crown-rump length (mm) | 108.1±3.8 | 115.9±5.7 |
|  | Paired gonad weight (mg) | 32.6±2.9 | 35.1±5.7 |
|  | Anogenital distance (mm) | 9.7±0.7 | Data available for <50% of fetuses |
| Maternal | Age (years) | 25.7±1.0 | 25.9±1.4 |
|  | BMI | 25.4±0.8 | 22.1±0.6 |
| **Fetuses used for testis qPCR** | | | **Fetuses used for testis steroids** |
| Characteristics | | *n*=22 | *n=25* |
| Fetal | Weeks of gestation | 14.9±0.5 | 13.6±0.2 |
|  | Body weight (g) | 89.6±17.9 | 45±3.2 |
|  | Crown-rump length (mm) | 100.0±7.0 | 88.4±1.9 |
|  | Paired gonad weight (mg) | 36.0±5.3 | 19.2±1.5 |
| Maternal | Age (years) | 24.3±1.2 | 24.2±1.3 |
|  | BMI | 25.2±1.5 | 24.7±1.1 |
| **Fetuses used for adrenal gland qPCR** | | | **Fetuses used for adrenal gland steroids** |
| Characteristics | | *n*=21 | n=30 |
| Fetal | Weeks of gestation | 15.0±0.5 | 15.2±0.7 |
|  | Body weight (g) | 89.4±15.8 | 94.8±13.7 |
|  | Crown-rump length (mm) | 102.6±6 | 103.3±5.6 |
| Maternal | Age (years) | 24.8±1.4 | 24.7±1.0 |
|  | BMI | 26.1±1.5 | 25.4±1.1 |
| **Fetuses used for liver qPCR analyses** | | | **Fetuses (from HDBR) used for liver steroids** |
| Characteristics | | *n*=45 | *n=20* |
| Fetal | Weeks of gestation | 14.0±0.3 | 13±0.6 |
|  | Body weight (g) | 66.8±10.2 |  |
|  | Crown-rump length (mm) | 94.4±4.4 | 133±2.1 |
|  | Paired gonad weight (mg) | 25.5±3.2 |  |
| Maternal | Age (years) | 23.7±1.3 |  |
|  | BMI | 25.7±1.0 |  |
| **Fetuses (from HDBR) used for placenta qPCR and steroids** | | |  |
| Characteristics | | *n*=20 |  |
| Fetal | Weeks of gestation | 13±0.6 |  |
|  | Crown-rump length (mm) | 133±2.1 |  |
| **Fetuses used for genital tubercle qPCR** | | |  |
| Characteristics | | *n=10* |  |
| Fetal | Weeks of gestation | 14.9±0.7 |  |
|  | Body weight (g) | 77.1±2.6 |  |
|  | Crown-rump length (mm) | 100.3±9.9 |  |
|  | GT weight (mg) | 23.7±9.2 |  |
|  | GT length (mm) | 4.0±0.3 |  |
| Maternal | Age (years) | 26.0±1.6 |  |
|  | BMI | 27.9±2.2 |  |
| **Fetuses used for testis incubations (Sweden)** | | |  |
| Characteristics | | *n=4* |  |
| Fetal | Weeks of gestation | 10.6±0.4 |  |
| Maternal | Age | 24.3±1.8 |  |
|  | BMI | 30.1±4.8 |  |

| **Fetuses used for gonad steroid hormone determination by GC-MS/MS** | | | |
| --- | --- | --- | --- |
| Characteristics | | Male n=6 | Female n=4 |
| Fetal | Weeks of gestation | 17.2±0.8 | 17.3±1.0 |
|  | Body weight (g) | 15.7±5.7 | 17.0±3.5 |
|  | Crown-rump length (mm) | 127.5±6.2 | 124.0±12.0 |
|  | Paired gonad weight (mg) | 45.2±2.7 | 35.7±7.6 |
|  | Anogenital distance (mm) | 13.6±1.2 | 7.3±1.3 |
| Maternal | Age (years) | 23.3±2.3 | 24.5±3.0 |
|  | BMI | 25.7±2.2 | 28.4±4.5 |
