# Supplementary material for: Alternative (backdoor) androgen production and masculinization in the human fetus
Source: PLoS Biol. 2019 Feb 14;17(2):e3000002. doi: 10.1371/journal.pbio.3000002 (PMC6375548; doi:10.1371/journal.pbio.3000002)
Supplement: S4 Table — qPCR, quantitative PCR. (DOCX) [file pbio.3000002.s008.docx]

Primers used for qPCR

Gene Genbank Primers

*AKR1C2* NM_001354 ctgtgagggaggaagaaacatttgctaa

ggcttctattgccaatttgacggc

*AKR1C3* NM_003739 tccgaccagccttggaaaactca

cccaggtggtacagagatccactatgtc

*AKR1C4* NM_001818 gctgtagaggtcaccaaattagcaatagaagc

gtggttgaaagaaagtgcaccaaagct

*CYP17A1* NM_000102 cccatctattcggttcgtatgggc

gccagagtcagcgaaggcga

*HSD3B1* NM_000862 gaggatcatccgcctcttggtgaag

ccagcactgtcagcttggtct

*HSD3B2* NM_000198 gaggatcgtccgcctgttggtggaa

cttcaagtacagtcagcttggtcc

*HSD17B3* NM_000197 ccatttcctgaacgcaccgg

agagaggccaaggaaacagggct

*HSD17B6* NM_003752 tctgacaggctggagacggtgac

gtgttcagccactcacataaggtaattgg

*SRD5A1* NM_001047 catgttcctcgtccactacgggc

cagcatacactgcacaatggctca

*SRD5A2* NM_000348 cccctctccctcttcgggc

aggactccatttccagtgcagaagg

*TBP* NM_003194 aggaaaaaattgaatagtgagacgagttcca

tggactaaagatagggattccgggagt

Primers used for sexing fetuses

Gene Genbank Primers

*ZFX* NM_003410 gcacttctttggtatctgagaaagt

ataatcacatggagagccacaagct

*SRY* NM_003140 cccatgaacgcattcattgtgtgg

attttagccttccgacgaggtcgata
